# Evaluation of a quantitative RT-PCR assay for the detection of the emerging coronavirus SARS-CoV-2 using a high throughput system

**DOI:** 10.2807/1560-7917.ES.2020.25.9.2000152

**Published:** 2020-03-05

**Authors:** Susanne Pfefferle, Svenja Reucher, Dominic Nörz, Marc Lütgehetmann

**Affiliations:** 1 Institute of Medical Microbiology, Virology and Hygiene, University Medical Center Hamburg-Eppendorf (UKE), Hamburg, Germany

**Keywords:** SARS-CoV-2, COVID-19, high-throughput PCR, automation, molecular diagnostic

## Abstract

Facing the emergence of severe acute respiratory syndrome coronavirus 2 (SARS-CoV-2), high-volume respiratory testing is demanded in laboratories worldwide. We evaluated the performance of a molecular assay for the detection of SARS-CoV-2 on a high-throughput platform, the cobas 6800, using the ‘open channel’ for integration of a laboratory-developed assay. We observed good analytical performance in clinical specimens. The fully automated workflow enables high-throughput testing with minimal hands-on time, while offering fast and reliable results.

In January 2020, a previously unknown coronavirus – now named severe acute respiratory syndrome coronavirus 2 (SARS-CoV-2) – was identified as causative agent of a cluster of suspicious pneumonia cases in Wuhan, China [1,2]. The World Health Organization (WHO) declared a public health emergency of international concern by the end of January 2020 [3]. As at 3 March 2020, more than 90,000 confirmed cases and more than 3,100 fatalities in 13 countries have been attributed to the virus.

The ability to quickly confirm or clear suspected cases is crucial during global outbreak scenarios, especially when clinical manifestations are difficult to distinguish from other respiratory infections such as influenza, molecular diagnostics is key for detection of the emerging virus. A variety of suitable assays were made available early on during the course of the outbreak, notably by Corman et al. and others [4,5]. However, their implementation in the diagnostics laboratory usually relies on manual PCR setups requiring a high degree of human interaction for execution and interpretation, thus limiting their capacity to be scaled up for handling large numbers of samples. 

In this study we report the analytical evaluation of a laboratory-developed test for the detection of SARS-CoV-2 using the open channel (utility channel) of the cobas 6800 system.

## SARS-CoV-2 Utility Channel test setup

A custom-made primer/probe set based on a previously published assay targeting the E gene [4] was optimised for the use on the automated system. Primer and probes were ordered from IDT DNA Technologies (Coralville, United States (US)). Both primers were modified with 2’-O-methyl bases in their penultimate base to prevent formation of primer dimers. The ZEN double-quenched probe (IDT) was used in order to lower background fluorescence. The master mix (Mmx) cassette (for 96 tests) is prepared by combining 84 µL forward primer (400 nM, 5´-ACAGGTACGTTAATAGTTAATAGCmGT-3´), 84 µL reverse primer (400 nM, 5´-ATATTGCAGCAGTACGCACAmCA-3´) and 10.5 µL probe (50 nM, 5´-Fam-ACACTAGCC/ZEN/ATCCTTACTGCGCTTCG-Iowa Black FQ-3’)* together with 182 µL water and 5,640 µl Mmx2 mixture. After mixing, the 6 mL are transferred to the reagent cassette. The Mmx cassette is delivered with a full-process control: it is preloaded with an internal control (IC) RNA together with primers and probe of the IC detection assay by default [6]. Instrument settings in the cobas mni Utility Channel software (Roche, Los Gatos, US) and the temperature profile used for the RT-PCR reaction are summarised in Table 1.

**Table 1:** SARS-CoV-2 PCR cycling conditions and software settings in the Utility Channel software used to create the run template

Sample type	Alcohol-based swab (400 µL input)				
Channels	1	2:*SARS-CoV-2 E-gene*	3	4	IC
RFI	NA	1.5	NA	NA	Predefined
PCR cycling conditions	UNG incubation	Pre-PCR step	1st measurement	2nd measurement	Cooling
Number of cycles	Predefined	1	5	45	Predefined
Number of steps		3	2	2	
Temperature		55 °C; 60 °C; 65 °C	95 °C; 55 °C	91 °C; 58 °C	
Hold time		120 s; 360 s; 240 s	5 s; 30 s	5 s; 25 s	
Data acquisition		None	End of each cycle	End of each cycle	

IC: internal control; NA: not applicable; RFI: relative fluorescence increase; UNG: uracil-DNA N-glycosylase.

Assay performance was evaluated for swab samples. All clinical specimens used were collected at the University Medical Center Hamburg-Eppendorf (UKE-HH). Samples were mixed 1:1 with Roche cobas PCR media (≤ 40% guanidine hydrochloride in Tris-HCL buffer) and incubated for 30 min before loading onto the cobas 6800 system. Apart from that sample preparation step, no further manual steps are required during the entire workflow of the novel assay.

## SARS-CoV-2 Utility Channel test performance evaluation

**Figure 1:**
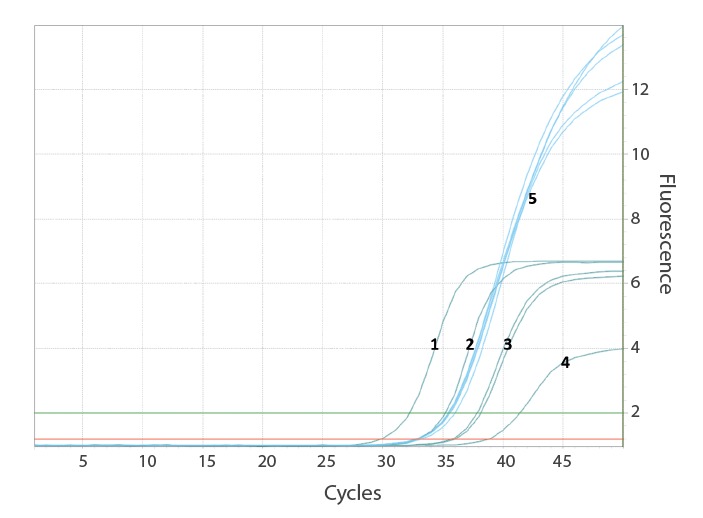
Example for amplification curves of the SARS-CoV-2-UCT SARS-CoV-2 Utility Channel test

**Figure 2:**
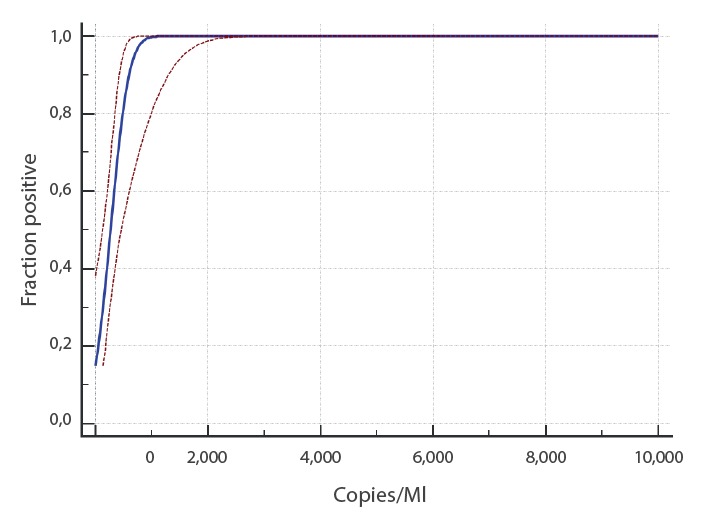
Determination of limit of detection of the SARS-CoV-2 Utility Channel test based on in-vitro transcribed RNA

Limit of detection (LoD), inter-run variability and cross-reactivity with other respiratory pathogens were determined. In-vitro transcribed RNA (IVT RNA) of the E gene of SARS-CoV-2 and purified RNA of SARS-CoV (strain Frankfurt-1) were used as positive controls (obtained via the European virus archive global (EVAg), https://www.european-virus-archive.com) [7]. Limit of detection of the SARS-CoV-2 UCT was determined by analysing each of eight replicates of a dilution series containing IVT RNA diluted in E-swab medium (Copan, Brescia, Italy; modified liquid Amies medium) and Roche cobas PCR medium (1:1) at 10,000, 1,000, 500, 250 and 125 copies/mL and eight negative samples. The LoD was 689.3 copies/mL with 275.72 copies per reaction at 95% detection probability (Figure 1, Figure 2). For estimation of inter/intra-run variability, we analysed each of two concentrations (ca 5 × and 10 × LoD spiked IVT SARS-CoV-2 RNA) in five replicates and a negative sample with five replicates in two runs each. Minimal deviation was observed with ± 0.5 cycle threshold (Ct) at 10 × LoD and 0.75 Ct at 5 × LoD. No false positive results occurred.

IVT RNA: in-vitro transcribed RNA.

Dark blue curves: specific SARS-CoV-2 signals of IVT-RNA at 100,000 copies/mL (1), 10,000 copies/ml (2), 1,000 copies/ml (3) and 100 copies/ml (4). Light blue curves: signals of the internal control (5).

IVT RNA: in-vitro transcribed RNA.

Probit analysis revealed a limit of detection of 689.3 copies/mL at 95% probability. Blue line: probit curve (dose-response rule); red dotted lines: 95% confidence intervals. IVT-RNA obtained by EVAg.

Potential interference of the SARS-CoV-2 UCT with other respiratory pathogens (including other human CoV strains) was evaluated by analysing 88 previously determined clinical samples and an external quality control assessment panel (INSTAND, Düsseldorf, Germany) containing lysates of infected cells. None of these organisms were detected by the SARS-CoV-2 UCT assay (Table 2), confirming high specificity of the assay for viruses within the *Betacoronavirus* subgenus *Sarbecovirus* [4].

**Table 2:** Potential cross-reactivity SARS-CoV-2 Utility Channel test with other respiratory pathogens, evaluated with a panel of organisms typically found in respiratory infections

Clinical samples with known viruses	Number tested
Coronavirus (not typed)	5
hCoV HKU-1	2
hCoV NL63	1
Adenovirus	2
Bocavirus	7
Human metapneumovirus	6
Influenza A	7
Influenza A(H1N1)	6
Influenza B	3
Parainfluenza 1 virus	3
Parainfluenza 2 virus	1
Parainfluenza 3 virus	8
Parainfluenza 4 virus	3
Respiratory syncytial virus (A/B)	10
Rhino/enterovirus	8
*Chlamydophila pneumoniae*	4
*Mycoplasma pneumoniae*	4
*Legionella pneumophila*	3
*Bordetella pertussis*	4
*Bordetella parapertussis*	1
**Total number of clinical samples**	**88**
External quality control assessment panel with known viruses (lysates of infected cells)	
hCoV 229E	1
hCoV NL63	1
hCoV OC43	3
MERS-CoV	4
Coxsackievirus A21	1
Coxsackievirus B3	1
Enterovirus 68	1
Rhinovirus	3
Human metapneumovirus	3
Virus-negative	4
**Total number of quality control assessment panel samples**	**22**
**Total number of samples**	**110**

hCoV: human coronavirus;

Clinical samples had been pre-analysed by routine diagnostic. External quality control assessment panel (INSTAND) samples contained inactivated lysates of virus infected cells including four virus-negative cell lysates.

## Discussion

Outbreaks of novel pathogens, such as the ongoing SARS-CoV-2 situation, represent a challenge for molecular diagnostics. However, identification of the agent, sequencing and publication of specific PCR assays for general use, a process that took several weeks 15 years ago, can now be accomplished in a few days [8]. With this rapid pace in identification and sharing of information comes the responsibility of local laboratories to be able to implement available assays and provide tests for the outbreak strain in an equally short timeframe. Furthermore, demand can be unpredictable and may suddenly spike, even outside the primary endemic areas, putting testing capacity under strain and potentially causing delays. For example, in February 2020, an entire cruise ship carrying almost 4,000 passengers and crew was quarantined off the port of Yokohama, Japan, and hundreds of people had to be tested for the virus in a short period of time [9]. 

Automated solutions for molecular diagnostics can help handle large numbers of samples and can be scaled to keep pace with fluctuating demand [10-12]. The system used in this study fully automates nucleic acid extraction, purification, amplification and detection. We, among others, have previously demonstrated that laboratory-developed tests can be adapted for fully automated PCR platforms such as the cobas 6800 system [6,13]. After brief preparation, clinical samples can be loaded directly into the device; required hands-on time and manual steps are reduced by up to 60% (manual steps reduced from 33 to 14, hands-on time reduced from 74 min to 14 min) compared with conventional workflows which usually involve automated extraction and PCR performed as separate procedures [13]. The inclusion of a full-process control for each reaction further facilitates the handling of results, allowing interpretation by personnel not familiar with RT-PCR diagnostics.

The system has passed clinical evaluation for a variety of viral and bacterial targets [14-17] and is also used for blood safety testing, another diagnostic field in which large numbers of samples have to be cleared by PCR-based screening tests.

In this study, we demonstrated good analytical performance of an adapted SARS-CoV-2 assay on swab samples with an LoD of 689.3 copies/mL (e.g. 275.72 copies/process) at 95% detection probability, which is roughly in line with results published by Corman et al. [4]. They report an LoD for SARS-CoV-2 RNA of 5.2 copies per reaction at 95% detection probability, which corresponds to 208 copies/mL based on a 25 µL reaction volume. It has to be noted that manual determination of LoD usually involves adding purified target RNA directly into the reagent mix for amplification, whereas in our study, control material (purified RNA) was spiked into samples and underwent the full workflow of the cobas 6800 device, including extraction and purification. Therefore, differences in nominal analytical performance are to be expected. One important limitation of the study is that we had to rely on spiked-in material and could not validate the performance of the assay using clinical SARS-CoV-2-positive samples.

However, we believe that our assay designed for high-throughput molecular testing could be useful in the ongoing outbreak situation. It demonstrates how commercial PCR platforms can enhance outbreak readiness for emerging pathogens, allowing for large numbers of patients to be screened in a reasonable timeframe, if necessary. It has to be noted that by its nature as a screening test targeting only a single viral gene, positive results should always be confirmed with an independent PCR as recommended [4]. Finally, we want to stress the importance of closely coordinating with local reference centres and public health authorities for determining clinical indications for testing, as well as the handling of confirmed cases and contact precautions.

## *Author’s correction

In the original version of this article published on 5 March 2020, a wrong sequence was presented for the probe and quencher (5´-Fam-ACACTAG/ZEN/ CCATCCTTACTGCGCTTCG-BHQ-3´). At the request of the authors, this was corrected to read 5´-Fam-ACACTAGCC/ZEN/ATCCTTACTGCGCTTCG-Iowa Black FQ-3’. This change was made on 11 March.
