# Authors’ correction for Euro Surveill. 2020;25(9)

**DOI:** 10.2807/1560-7917.ES.2020.25.10.20200312c

**Published:** 2020-03-12

**Authors:** Eurosurveillance editorial team

**Affiliations:** 1 European Centre for Disease Prevention and Control (ECDC), Stockholm, Sweden

In the article ‘Evaluation of a quantitative RT-PCR assay for the detection of the emerging coronavirus SARS-CoV-2 using a high throughput system’ by Pfefferle et al. published on 5 March 2020, a wrong sequence was presented for the probe and quencher (5´-Fam-ACACTAG/ZEN/ CCATCCTTACTGCGCTTCG-BHQ-3´). At the request of the authors, this was corrected to read 5´-Fam-ACACTAGCC/ZEN/ATCCTTACTGCGCTTCG-Iowa Black FQ-3’. This change was made on 11 March.

